# Development and item selection of the language and dyslexia screening questionnaire in primary care settings

**DOI:** 10.3389/fped.2026.1750893

**Published:** 2026-05-13

**Authors:** M. Adelaida Restrepo, R. J. Risueño, Jeffrey M. Williams, Shelley Gray, Carla C. Allan, Nilam Patel Khurana, Jodi Carter, Rebecca Keller, Kathleen Swartz, Terrence Stull, Ryan Seltzer, Savannah H. Romeo

**Affiliations:** 1 Department of Communication Sciences and Disorders, University of South Florida, Tampa, FL, United States; 2 College of Health Solutions, Arizona State University, Tempe, AZ, United States; 3 Phoenix Children’s Hospital, Phoenix, AZ, United States; 4 Department of Child Health, College of Medicine, University of Arizona, Phoenix, AZ, United States; 5 Healing Hearts Pediatrics, Mesa, AZ, United States; 6 Department of Pediatrics, Valleywise Health, Phoenix, AZ, United States; 7 Learning to Soar, Gilbert, AZ, United States

**Keywords:** developmental language disorder (DLD), dyslexia, parent questionnaire, screening, well-check visit

## Abstract

**Purpose:**

The study's purpose was to develop and select items for a caregiver-completed screening tool to assess children for dyslexia and developmental language disorder (DLD).

**Method:**

Participants were recruited from community centers, schools and pediatric offices. Caregivers clicked a QR code on a flyer to access the consent and questionnaire, or contacted the research assistant to access them, and completed the screening measure designed to assess the risk of DLD and dyslexia. Then, children completed a gold-standard battery that included language, reading, phonological awareness, rapid naming, and memory measures.

**Results:**

Participants included 149 children and their caregivers. We used unadjusted logistic regressions to estimate the predictive value of each individual survey question for dyslexia, DLD, and typical development (TD) to retain questions at *p* < 0.05. Then, three stepwise logistic regressions were run to obtain a final, restricted model using the outcomes of dyslexia, DLD, and TD vs. any other diagnosis. Analyses indicated that the best-fitting model predicting TD vs. other groups in the combined sample included 14 items. The AUC for this set was 0.84. The specificity and sensitivity were 0.69 and 0.80, respectively. For the monolingual and bilingual samples separately, AUCs and sensitivity values were all greater than 0.80, and specificity values were 0.69 or above.

**Conclusions:**

The findings indicate that the 14-item screener may effectively identify children at risk for dyslexia and DLD. Further validation, including the development and testing of additional items in a large, representative sample, is needed to establish its validity at the population level.

## Introduction

The Bright Futures national health promotion and prevention initiative, led by the American Academy of Pediatrics with support from the US Department of Health and Human Services, is designed to foster the implementation of evidence-based well-child and adolescent care within the medical home and beyond via partnerships with community organizations, school-based health centers, and state agencies (1). Indeed, a Bright Futures introductory video states, “Promoting Children's Health is Everybody's Business.” While primary care pediatricians have a long, rich history of screening for developmental delays in early childhood, dyslexia and developmental language disorder (DLD) are not, and are often unrecognized, highly prevalent conditions present in school-aged children (5%–10% and 7.5%, respectively) (2–6). These conditions are associated with school failure and increased risk for unemployment, underemployment, and incarceration (7, 8). Early identification of these conditions should be a priority in health and education.

DLD is a significant impairment in the ability to understand and produce spoken language despite otherwise typical development (9). Dyslexia is defined as a specific impairment of word reading and/or spelling and is often accompanied by difficulties in phonological processing and oral language weaknesses (10). DLD and dyslexia co-occur frequently and early identification and intervention ameliorate their effects and prevent later negative academic, social, and health effects (11). For example, Lovett et al. found that those receiving intervention in first and second grade made twice as many gains as those who first received intervention in third grade (11). Over 40 states require schools to conduct dyslexia screening for elementary school children (12), but none require screening for DLD (2).

There is an urgent, unmet need for efficient, cost-effective screening that could improve the early identification and intervention for at-risk children; building redundancies in systems of care would be one promising step forward, much in the way that schools and primary care pediatric providers (PCPs) screen for visual and auditory impairments. Additionally, this 2-systems approach aligns with the American Academy of Pediatrics (AAP) emphasis on promoting literacy from birth (13).

Current Bright Futures guidelines recommend that PCPs screen for broad academic performance, but do not yet include expert-driven language and literacy assessment recommendations that are sufficiently actionable for the PCP and effectively link back to the school setting (i.e., advocacy strategies and/or implementation guidance once children are identified as at-risk). Bright Futures guidelines include a list of recommended screening assessments tailored to each age group. For the six-year-old well-child visit, the Strengths and Difficulties Questionnaire (14) is a caregiver-administered tool that primarily focuses on behavior and social skills, but it does not assess oral language or early literacy development. Another tool is the Parents’ Evaluation of Developmental Status—Revised (PEDS-R) (15). The PEDS-R includes one open-ended question about expressive language (e.g., vocabulary, articulation, syntax, vocal quality) and one open-ended question about receptive language (e.g., understanding sounds, words, gestures, answering why-questions, drawing inferences). It also features a ’school’ question addressing letter knowledge, sounding out words, spelling, and vocabulary. However, caregivers must fully understand the open-ended questions to accurately assess risk, and most studies of the PEDS-R have focused on younger children. Without a validated and accessible screening tool in the clinical setting, data indicates that physicians have difficulty identifying developmental or behavioral problems (16).

## Screening for literacy disorder risk in pediatric clinical visits

Aside from Bright Futures, two tools have been developed to screen children at-risk for literacy disorders during pediatrician check-ups. Indeed, a caregiver questionnaire (17) and an interactive book reading measure (18) have demonstrated predictive validity for identifying preschoolers at risk for literacy difficulties. However, these measures were designed for children aged 3–5 years, limiting their applicability to school-aged populations—a critical window for linkage to effective and efficient interventions within the school setting.

## Caregiver report to identify risk of DLD

Several studies have found that caregiver report can identify risk of DLD (9, 19–21). Ebert et al. found that caregiver report can accurately identify risk of DLD and contributes to the accuracy of diagnosis, although other studies have found that caregiver report alone is not accurate (20, 22). Further examination is needed to determine whether caregiver report can indicate risk of DLD, rather than diagnosis, with additional questions that target specific current language behaviors and family history of speech and language difficulties. Although investigators have found no predictive value of caregiver report on identifying DLD, (23) Hendricks et al. found that the caregivers expressed more concerns about language and reading difficulties in their child and a family history of special services than those caregivers with typically-developing children (22). These studies suggest that caregiver report can be used for screening purposes.

Caregiver report has also been found to help identify risk of DLD in diverse learners (24). For example, Restrepo found that emergent bilinguals in preschool, kindergarten, and first grade could be identified based on caregiver report of speech and language difficulties, family history, and language sampling; however, the measure demonstrated high specificity, but low sensitivity (21). Similarly, the Alberta Language and Development Questionnaire (ALDeQ) was developed as a caregiver report evaluating the multilingual child's home and school language skills relative to those with typical language in Canada (25), and it, too, showed low sensitivity and high specificity. In contrast, Pratt et al. found that a combination of teacher and caregiver reports yielded high sensitivity and low specificity (26). Abutbul-Oz and Armon-Lotem found that a caregiver questionnaire had high accuracy in identifying DLD in bilingual children in Israel (24). Although these measures may prove to be effective in identifying multilingual children with DLD, these are not appropriate for well-child visits because they are long, and for screening purposes in medical offices, they need to be relatively short and user-friendly. To this effect, Auza et al. found that when caregivers of six-year-old children expressed concerns on four questions from Restrepo the likelihood that the child presented with DLD tripled (19, 21).

## Caregiver report for risk of reading difficulties

Bryant et al. found that caregiver concern about reading difficulties in second graders was correlated with the presence of reading difficulties in these children (27). The specificity of the measure was 0.81, although the sensitivity was 0.59. In addition, children whose caregivers did not express concerns about their reading skills had better word-reading skills than those whose caregivers did. These results suggest that caregivers’ reports may be accurate and indicative of children who are at greater risk or those with more severe difficulties. Patrick et al. found that caregiver report was accurate in identifying typical reading and math learning; (28) however, their sensitivity or identification of reading difficulties and math difficulties based on caregiver reports was fair. These studies suggest that further work in caregiver reports is still needed to ensure the measures identify the risk of DLD and dyslexia efficiently during well-check visits.

## Study purpose

The current project aimed to develop a caregiver-completed screening tool to identify risk factors for dyslexia and DLD during six-year well-child visits in pediatric primary care settings. Given the high comorbidity between dyslexia and DLD, (29) the screening measure was designed to identify children at risk for either disorder. Our goal is to support early risk identification and referral for comprehensive assessment and intervention, rather than diagnosis. The current study describes the development phases of the Language and Dyslexia Screening Questionnaire (LDYSQ). The LDYSQ was developed in four phases: (1) item development by a multidisciplinary team that included pediatricians, speech-language pathologists, caregivers of children with dyslexia, and a reading tutor; (2) evaluation of caregivers’ interpretation of items and refinement based on their feedback; (3) comparison of questionnaire risk scores with performance on gold-standard assessments of dyslexia and DLD; and (4) identification of item sets that most strongly differentiated risk categories, thereby maximizing discriminant validity.

## Methods

### Phase I—item development

LDYSQ is a caregiver screening questionnaire designed to identify children at risk for dyslexia and DLD. Items were selected based on consensus from the research team, which included a pediatrician, a reading tutor, speech-language pathologists, and a clinical psychologist. We developed 64 questions based on research on both disorders. Specifically, the team reviewed multiple iterations of questions, initially focused on dyslexia, and later added the language component due to the high comorbidity of the two disorders and the need for differential diagnosis. We also had multiple conversations to make the items more caregiver-friendly. Item development was guided by a content framework derived from the literature on dyslexia and developmental language disorder, including domains related to family history, early language development, phonological processing, letter–sound knowledge, educational history, and prior services. Items were reviewed iteratively by the study team, and consensus regarding item inclusion and wording was reached through group discussion rather than formal Delphi procedures.

Items focused on family history (i.e., familial presence of a language, reading, or language learning disability), educational history (i.e., current grade in school, preschool attendance, tutoring, outside help), language, speech, and reading behaviors (i.e., “My child was a late talker”, “My child leaves off the ending sounds of some words”, and “My child has difficulty remembering the names of letters”), and medical history (e.g., vision or hearing impairments, specific learning disabilities, developmental delays, medical diagnoses, behavioral or emotional disorders, etc.). For most items, the response set included ‘*Yes,’* ‘*No,’* or ‘*I don't know*;’ however, some items within the developmental history section included the response option ’Sometimes.’ The questionnaire was developed in English and subsequently adapted for use in Spanish.

### Phase II—caregiver feedback

After developing the initial questionnaire, we engaged eight caregivers—four English-speaking and four Spanish-speaking—to share their understanding of each item and its appropriateness for caregivers. We ran two focus groups in English. The Spanish focus groups were conducted individually due to scheduling constraints. Caregiver feedback was obtained using a structured qualitative approach. Caregivers were asked to describe their interpretation of each item in their own words and to indicate whether wording was clear, confusing, or potentially ambiguous. Feedback was documented by the session leader and reviewed by the full research team. Items were revised to improve clarity, add behavioral examples, or reduce ambiguity, and items judged to be redundant or difficult to interpret were eliminated through team consensus.

### Phase III and IV—testing with gold standard measures

#### Participants

This phase of the study was designed as an exploratory, early-phase investigation to inform item selection and preliminary performance characteristics of the LDYSQ. The Arizona State University Institutional Review Board approved this study. It was carried out in the Phoenix, Arizona, and Tampa, Florida metropolitan areas. We distributed recruitment flyers to pediatric offices, schools, and community centers throughout the metropolitan area. All families were compensated $25 for their time. Recruitment flyers stated that our team was looking for six-year-old children and their caregivers to help us develop a dyslexia and language disabilities screening measure. Due to the preliminary nature of this study, we recruited a convenience sample.

Participants had to (a) be six years of age, (b) reside in a home where English or Spanish was the primary language per caregiver report, (c) have normal hearing and normal or correctable vision, and (d) have normal neurocognitive development per caregiver report (e.g., no global developmental delay, or any other neurological impairment that would interfere with testing). Due to the high rates of comorbidity of DLD and attention deficit hyperactivity disorder (ADHD), (30, 31) ADHD was not deemed an appropriate exclusionary criterion of our study. All study participants passed an initial hearing screening and had normal or correctable vision. One caregiver reported that the child had Autism spectrum disorder, and three caregivers reported that their child had emotional disturbances. We eliminated that child with autism because this child would not meet either DLD or dyslexia criteria. See the procedures for a description of the recruitment protocol.

Tables 1, 2 present the mean scores for each standardized decoding and oral language measure administered to the monolingual and bilingual groups, respectively. Most participants in the monolingual group were female first graders (*n* = 56, 50.5%), with an average age of 77.8 months (SD = 3.81). Participants in the bilingual group were primarily male (*n* = 22, 57.9%) first graders with an average age of 78.0 months (SD = 3.65). Most participants were recruited from the Phoenix metropolitan area across both the monolingual (*n* = 109, 98.2%) and bilingual (*n* = 26, 68.4%) groups. The rest were recruited from the greater Tampa, FL area through schools and community organizations.

**Table 1:** English monolingual participant means (SDs) on standardized measures.

Measure	N	Mean	SD
KBIT-2 Matrices Subtest Standard Score	111	98.8	14.8
CTOPP-2 Phonological Awareness Composite Score	111	98.4	16.4
CTOPP-2 Phonological Memory Composite Score	110	98.9	14.2
CTOPP-2 Rapid Symbolic Naming Composite Score	111	99.6	15.1
CTOPP-2 Rapid Non-Symbolic Naming Composite Score	110	97.9	18.8
CELF-5 Core Language Composite Standard Score	111	100	14.5
WRMT-3 Basic Skills Cluster Score	111	99.8	16.2

CELF-5, Clinical Evaluation of Language Fundamentals fifth edition; CTOPP-2, Comprehensive Test of Phonological Processing second edition; KBIT-2: Kaufman Brief Intelligence Test second edition; WRMT-3, Woodcock Reading Mastery Tests third edition.

**Table 2:** Spanish-english bilingual participant means (SDs) on standardized measures.

Measure	N	Mean	SD
KBIT-2 Matrices Subtest Standard Score	38	97.7	16.0
CTOPP-2 Phonological Awareness Composite Score	31	92.3	14.1
CTOPP-2 Phonological Memory Composite Score	31	92.8	17.6
CTOPP-2 Rapid Symbolic Naming Composite Score	31	96.8	10.3
CTOPP-2 Rapid Non-Symbolic Naming Composite Score	30	94.5	13.8
WRMT-3 Basic Skills Cluster Score	32	95.7	14.9
BESA Language Index Score	38	104	11.1
TOPPS First Sound Raw Score	38	7.21	2.45
TOPPS Memory for Digits Raw Score	38	6.84	1.97
TOPPS Blending Words Raw Score	36	10.1	3.96
TOPPS Segmenting Words Raw Score	35	5.89	3.86

CELF-5, Clinical Evaluation of Language Fundamentals fifth edition; CTOPP-2, Comprehensive Test of Phonological Processing second edition; KBIT-2: Kaufman Brief Intelligence Test second edition; WRMT-3, Woodcock Reading Mastery Tests third edition.

### Procedures

Recruitment flyers were posted or disseminated in community centers (e.g., libraries, centers for immigrant families), schools, and pediatric offices. We recruited physicians through meetings and emails where they had a link to a short video that explained the disorders and how the process would work. If families were interested, they used the QR code to access additional information, signed the consent, and then completed the questionnaire. For families that could not use the QR code, they emailed or called/texted the phone number to contact us. Once the caregiver consented to their child's participation, they completed the LDYSQ online or in person with the research assistant. Within two weeks of completing the consent form and LDYSQ, their child underwent an in-person evaluation at a community location convenient for the family. Assessments were administered by graduate and undergraduate speech-language pathology research assistants (RAs) who were trained and supervised by a licensed speech-language pathologist. Each RA was required to meet 90% administration fidelity over two directly supervised administrations of each measure before administering measures independently. Fidelity was measured using a procedural checklist that evaluated each RA's ability to follow the order of assessments and administer assessments correctly. RAs were blinded to the children's classification, parent concern, or answers to the questionnaire. During the evaluation, children completed a hearing screening at 500, 1,000, 2,000, and 4,000 Hz in each ear individually at 20 dB, and gold-standard assessments to determine whether the child met criteria for dyslexia, DLD, or both. Testing took between one to two hours to complete for monolingual children and 1.5–2.5 for bilingual children. Children were given breaks or scheduled for two appointments to ensure that they were not too tired to complete the assessments. Each participant's family received a report that included a summary statement, accompanying scores, and whether the child was deemed at risk for dyslexia, DLD, or both, or was not at risk for either. For children at risk, families were given additional information to follow up with the schools.

### Measures

Children in the monolingual and bilingual groups completed the following assessments: the LDYSQ questionnaire, an English phonological processing measure—*Comprehensive Test of Phonological Processing, 2nd Edition* (*CTOPP-2*), (32) an English decoding task—*Woodcock Reading Mastery Test, 3rd Edition* (*WRMT-3*), (33) a rapid naming measures—from the CTOPP, and a nonverbal IQ screening—*Kaufman Brief-Intelligence Test, 2nd Edition* Matrices Subtest (*KBIT-2*) (34). In addition, the monolingual group completed the *Clinical Evaluation of Language Fundamentals, 5th Edition* (*CELF-5*) (35). The bilingual children were administered separate oral language—*Bilingual English Spanish Assessment* (*BESA*) (36) in Spanish and English and a Spanish phonological processing measures *Test of Phonological Processing in Spanish* (*TOPPS*) (37).

#### Language and dyslexia screening questionnaire (LDYSQ)

As described above, the questionnaire comprised 57 questions, including demographic and developmental information, as well as questions on pre-reading and oral language development. Caregivers of English monolingual participants took a median time of 5 min to complete the questionnaire, while caregivers of bilingual participants took a median time of 8 min.

#### Nonverbal IQ

Non-verbal IQ was screened using the *Kaufman Brief-Intelligence Test, 2nd Edition* Matrices Subtest (*KBIT-2*) (34). We included a nonverbal IQ measure because dyslexia and DLD are not associated with intellectual impairment. Children were not excluded based on non-verbal IQ scores given that these children may also be at risk of language and reading difficulties. The range of non-verbal IQ scores were similar for monolingual English-speaking (70–152) and Spanish-English bilingual participants (75–130).

#### Decoding

1. *Comprehensive Test of Phonological Processing, 2nd Edition* (*CTOPP-2*) (32)*.* The Phonological Awareness, Phonological Memory, Rapid Digit Naming (letters and numbers), and Rapid Object Naming (colors and objects) composites (M: 100 SD: 10) assessed children's pre-literacy skills. We used the standard scores based on age norms. The coefficient alpha was deemed acceptable at above 0.80 for all subtests except the Nonword Repetition, which consisted of an average alpha of 0.77.
2. *Woodcock Reading Mastery Test, 3rd Edition* (*WRMT-2*) (33). The Basic Skills Cluster (M: 100; SD: 10) assessed children's abilities to read real words and nonwords. We used the standard score based on age norms. Mean split-half reliability (alpha) for the Word Identification for early grades (pre-K–Grade 2) is 0.95.
3. *Test of Phonological Processing in Spanish* (*TOPPS*) (37)*.* Unfortunately, this measure had no norms. Nevertheless, we administered it, examined performance relative to all Spanish-speaking children in the group, and used it as part of the clinical judgment. Children in the no concern range were able to do the tasks in Spanish, whereas those with difficulty clearly could not pass the initial items. This performance was compared with the English measure for clinical judgement, while we also considered time spent in English and the number of schooling years.

#### Oral language

4. *Clinical Evaluation of Language Fundamentals, 5th Edition* (*CELF-5*) (35). Three subtests measuring children's vocabulary, morphology, and syntax formed the Core Language Composite Standard Score (M = 100; SD = 15). We used the standard score based on age norms. Coefficient alpha for children aged six was considered acceptable in English (0.93–0.95).
5. *Bilingual English Spanish Assessment* (*BESA*) (36). We administered the semantics and grammar subtests of the BESA to bilingual children. For those who spoke Spanish and English, we administered English and Spanish subsets of each modality and used the best score in any language for each domain to determine risk or no risk of DLD per test procedures and cut scores. Using assessments in both languages is the gold standard for bilingual children to ensure that there is no over- or under-estimation of language ability (36, 38, 39). For recent immigrants who only spoke Spanish, we administered only the Spanish BESA measures if they could not participate in any of the English testing. Coefficient alpha for children aged six was considered acceptable in Spanish (0.83–0.95) and English (0.75–0.95).

### Scoring reliability

Reliability was evaluated using internal consistency estimates and inter-rater agreement, where possible (Supplementary Table S1). Internal consistency was examined using Cronbach's alpha for all measures and subtests for which this approach was psychometrically appropriate. Alpha coefficients were generally high across measures, including the *KBIT-2* (*α* = 0.878, *n* = 149), *CTOPP-2* (*α* = 0.786–0.940, *n* = 139–144), *WRMT-2* (*α* = 0.923–0.938, *n* = 141–144), and *CELF-5* (*α* = 0.757–0.913, *n* = 110). Internal consistency was also high for bilingual-only assessments, including the *BESA* in both English (*α* = 0.893–0.936, *n* = 31–32) and Spanish (*α* = 0.921–0.964, *n* = 38), and the *TOPPS* (*α* = 0.726–0.877, *n* = 35–38).

Inter-rater reliability was assessed via double-scoring for a subset of participants. Agreement was high across measures, including the *KBIT-2* (ICC = 0.997, *n* = 12), *CTOPP-2* subtests (ICC = 0.867–1, *n* = 10–12), *WRMT-2* subtests (ICC = 0.996–0.998, *n* = 10–12), and *CELF-5* subtests (ICC = 0.919–1, *n* = 10–11). Inter-rater reliability for the bilingual-only assessments was unable to be calculated due to an insufficient number of double-scored participants. The number of double-scored participants was limited because the consent form did not specify permission for audio recordings, restricting the opportunities for independent scoring.

### Identification of dyslexia or DLD using gold standard tests

#### Dyslexia

**Figure 1:**
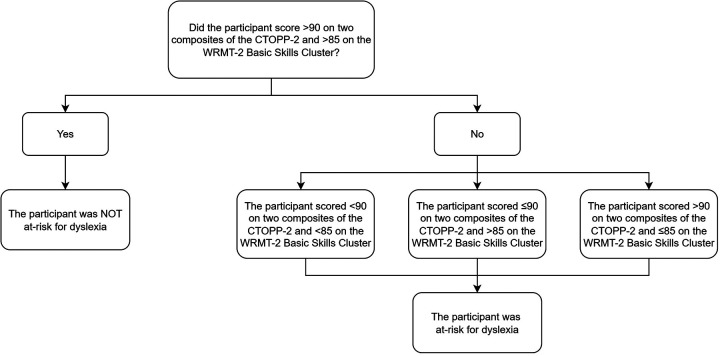
Decision tree for dyslexia at-risk determination for English monolingual participants.

Participants for the monolingual English-speaking sample met criteria for classification in the dyslexia group in one of the following ways (see Figure 1): (a) a standard score below 90 on one composite score of the *CTOPP-2* and a standard score below 85 on the Basic Skills Cluster of the *WRMT-2;* (b) a standard score below 90 on two composites of the *CTOPP-2* and a standard score of above 85 on the Basic Skills Cluster of the *WRMT-2,* or (c) a standard score of below 85 on the Basic Skills Cluster of the *WRMT-2* (the primary measure to identify decoding difficulties*)*. This ensures that we identify and reflect possible heterogeneous profiles of children at risk for dyslexia who need further diagnostic assessment.

Overall, dyslexia risk status for the bilingual sample was determined based on clinical judgment due to the lack of normed assessments. However, 9 of 23 children evaluated scored within the normal range on the English *CTOPP-2*, easing concerns of dyslexia risk. For bilingual participants, dyslexia risk status was determined using a structured clinical judgment process due to the absence of fully normed bilingual measures of decoding and phonological processing. Discrepancies were resolved through discussion to reach consensus.

#### Developmental language disorder

Participants in the monolingual English-speaking group met the criteria for classification in the DLD group if they achieved a scaled score of 85 or below on the Core Language Composite of the *CELF-5*. To meet the criteria for DLD, participants in the bilingual sample had to score below 90 on the Language Index of the BESA (We use the validated cut-scores for each measure, and therefore, cut scores are different across measures, which is best practice.)

## Results

### Phase II results

Eight parents participated in this phase. Based on their feedback, the study team clarified the wording of some questions and provided behavioral examples to enhance clarity. The caregivers primarily provided feedback on the vocabulary used in questions about specific disabilities (i.e., dyslexia, dyscalculia) and asked that we provide examples of the behaviors we were inquiring about (i.e., difficulty producing multisyllabic words; leaving the ends of words). We eliminated seven questions based on caregiver feedback and the research team's consensus of redundancy of questions or level of difficulty for caregivers, leaving a total of 57 questions.

### Phase III and IV general analysis strategy

The goal of the current study was to develop and identify a set of caregiver-administered items that show promise for identifying children at risk for dyslexia or DLD. The sample included 149 six-year-old children and one of their caregivers; 111 monolingual were English-speaking children and 38 bilingual Spanish-English speaking children. Our modest sample of participants precluded an item response theory (IRT) approach, so we needed a univariate approach to identify promising items. Although we could not conduct an IRT analysis, we could control for the non-independence of the two outcomes (dyslexia and DLD) when examining the predictive utility of each item by using a multilevel logistic analysis to estimate the odds ratios of each item simultaneously for both outcomes. Our sample consisted of two groups, one monolingual and one bilingual. We first developed the screener on the combined sample, then examined similarities and differences between the two.

Although the ultimate goal of this screener is to screen for Dyslexia or DLD, we needed to ensure that we included items that were predictive of each independently. Therefore, we used the following strategy. First, we selected the best predictors of dyslexia and DLD independently. This ensured that we started with an equal number of predictors for each outcome. Next, we combined these two sets and calculated a total score by summing the items together. Response options were coded as follows: “No” and “I don't know” = 0, “Sometimes” = 0.5, and “Yes” = 1. This approach was selected to preserve ordinal information while allowing partial endorsement of behaviors. Sensitivity analyses examining alternative codings are planned for future validation studies. Finally, we conducted three Receiver Operator Characteristics (ROC) analyses for each diagnosis individually and on the combined outcome of “Typically developing” vs. either diagnosis, which is the ultimate goal of this study. Using the above strategy, we ran the analyses several times, each time increasing the number of predictors selected from each list in step one and comparing the area under the curve (AUC) for each solution to determine the best set of items.

### Preliminary data examination

Our initial pool of items consisted of 46 unique items. However, when examining basic frequencies, four items received fewer than five affirmative responses (i.e., “Yes” or “Sometimes”) and were therefore dropped from future analyses due to the risk of inflated estimates and convergence problems. The correlation between dyslexia and DLD was 0.64, which justified the need for a multilevel analysis of item predictive power.

#### Identifying the best predictors

For the remaining 42 items, our first step in evaluating them was to run a multilevel logistic regression for each item on both outcomes simultaneously using the GLIMMIX procedure in SAS v9.4 and examine their odds ratios and *p*-values. For dyslexia, all items were in the expected direction; the odds ratios ranged from 1.05 to 19.53; and 28 items were significant at the 0.05 level. For DLD, all but two items had odds ratios in the expected direction; the odds ratios ranged from 0.57 to 19.15; and 12 of the items were significant at 0.05. The very high odds ratios were definitely a concern, but their estimates were reliable, and we felt that in this exploratory phase, their continued inclusion was justified. Statistical significance was used as an initial screening heuristic rather than as confirmatory evidence of item validity.

### ROC analyses of all three outcomes in the combined sample

Examination of the AUCs for all three outcomes revealed that selecting the top eight items for each outcome yielded the highest AUC value for identifying children at-risk for dyslexia or DLD. These two sets contained six items unique to each list and two items that appeared on both, yielding a final set of 14 items. The internal consistency of the 14-item total score was good (Cronbach's *α* = 0.8231). The AUCs for the total score calculated from these 14 items were 0.82, 0.80, and 0.84 for dyslexia, DLD, and dyslexia/DLD, respectively (see Table 3). For dyslexia, the AUC was 0.82 [SE = 0.04, 95% CI (0.74, 0.89)], and for DLD the AUC was 0.80 [SE = 0.05, 95% CI (0.70, 0.89)]. Table 3 presents the sensitivity and specificity of the measure across the range of values of the screener.

**Table 3:** Area under the curve, sensitivity, and specificity for each sample and combined.

Group	N	Performance metric	All vs. TD	DYS vs. TD	DLD vs. TD
Combined	135	AUC	0.84	0.82	0.80
		Sensitivity	0.80	0.78	0.78
		Specificity	0.69	0.65	0.66
Monolingual	103	AUC	0.83	0.82	0.73
		Sensitivity	0.77	0.75	0.75
		Specificity	0.69	0.66	0.57
Bilingual	32	AUC	0.87	0.78	0.95
		Sensitivity	0.80	0.88	1.0
		Specificity	0.73	0.63	0.88

AUC, area under the curve; DLD, developmental language disorder; DYS, dyslexia; TD, typical development. Confidence intervals for AUC, sensitivity, and specificity are reported in the results.

At the prespecified cutoff score of 3.5 for the combined dyslexia/DLD outcome, sensitivity was 0.69 [95% CI (0.59, 0.78)] and specificity was 0.80 [95% CI (0.68, 0.92)], which is below the 0.70 benchmark cited in American Academy of Pediatrics screening guidance. The positive predictive value was 0.87 [95% CI (0.80, 0.95)] and the negative predictive value was 0.56 [95% CI (0.44, 0.68)]. Model calibration for the combined outcome was adequate (Hosmer–Lemeshow *χ*² = 5.34, df = 6, *p* = 0.50). The overall misclassification rate for the combined model was 23%. At this cutoff, approximately 31% of children with dyslexia or DLD were not identified by the screener; however, performance estimates should be interpreted as optimistic, as item selection and evaluation were conducted within the same sample.

### Monolingual vs. Bilingual Sample

We initially enrolled 39 participants in the bilingual sample; however, three were dropped due to missing data and one was dropped for having an additional diagnosis of autism, leaving a sample of 35 children. We wanted to examine potential differential item functioning (DIF) between the two samples. To this end, we cycled through each of the 14 items selected for the screener. We then calculated the total score for the other 13 items (to eliminate any dependence between the total score and the outcome), and regressed the item score on a dichotomous indicator for the sample group, controlling for their level of at-risk as determined by the total score. Using this method, we found some evidence of DIF by group. The odds ratios for group difference ranged from −2.3 to 12.8 with an average of 1.3 in favor of the bilingual sample (meaning that the bilingual sample was more likely to endorse an item), but only two of the 14 estimates were reliable at *p* < 0.05. Furthermore, examination of the latter estimate revealed that fewer than five participants responded positively to that item, which can lead to inflated estimates, such as this. The next highest estimated difference was 4.0, but it was not reliable. These findings should be interpreted cautiously given the limited bilingual sample size.

AUC values for the monolingual sample were 0.83 for the combined dyslexia/DLD outcome [SE = 0.04, 95% CI (0.76, 0.91)], with sensitivity of 0.77 and specificity of 0.69 at the cutoff of 3.5. Model calibration for the monolingual combined model was adequate (Hosmer–Lemeshow *χ*² = 5.14, df = 7, *p* = 0.64).

AUC, sensitivity, and specificity for the two samples are shown in Table 3. These results indicate that for the bilingual group, items were slightly easier to endorse, but the differences were small and often unreliable.

### Sample size and missing data

For the multilevel logistic analyses of individual items, all available data were included from the 149 observations. Missing data varied from item to item, with a maximum missing of 10 participants for any given item. For the final models, 149 observations were read and 135 were retained for analysis. Fourteen cases were excluded due to missing questionnaire responses on the final 14 items or outcome classification. Given the small sample size, multiple imputation was not pursued.

## Discussion

The purpose of this study was to develop and provide preliminary evidence of feasibility of a caregiver screening tool to identify 6-year-old children at risk for dyslexia and DLD during the primary care well-child visit. During Phase I, we developed the questionnaire items as a multidisciplinary team, and in Phase II we evaluated caregivers’ understanding of the items and the acceptability of administration. Phase III findings indicated that the LDYSQ demonstrated preliminary accuracy for identifying children at risk for DLD and dyslexia. For the combined sample of monolingual English and Spanish-English bilingual participants, the area under the curve (AUC) for the combined dyslexia/DLD outcome was 0.84, with AUCs of 0.82 for dyslexia and 0.80 for DLD. In Phase IV, evaluation of individual screening items revealed six unique predictors of dyslexia and six unique predictors of DLD, with only two items overlapping between the disorders. Together, these findings suggest that the LDYSQ is a feasible early screening tool that warrants validation in a large, representative sample and may be appropriate for use during children's six-year well-child visits.

### Pediatricians’ role in identifying risk for language and literacy disorders

The American Academy of Pediatrics emphasizes the vital role of pediatricians in supporting early literacy skills; however, evidence-based screening measures practical for primary care remain limited (13). Commonly administered developmental tools, such as the Ages and Stages Questionnaire, do not assess emergent literacy skills and existing assessments are likely too time and resource-intensive to be feasibly administered during a 6-year-old well-child visit. The LDYSQ, once validated, shows promise for such purpose and has the potential to serve as a pivotal tool for identifying English- and Spanish-speaking children at risk for DLD and dyslexia in pediatric clinics during their 6-year well-child visit. This timeline aligns well with the opportunity for connections to additional school resources, which can profoundly benefit the early trajectory of learning for children with language and literacy disorders.

### Early efficacy of the LDYSQ

The LDYSQ demonstrates promising sensitivity and specificity relative to other early literacy screening measures, given further development and validation, including those developed for primary care settings (17). Further, the LDYSQ is more feasible than other such measures, enhancing the likelihood of broad implementation. Our results with 6-year-old children showed higher sensitivity (0.80) and specificity (0.69) than those reported by Iyer et al (17). A generally acceptable sensitivity is above 0.80, (40) which suggests early promise for our measure. At the same time, sensitivity varied across outcomes and did not consistently exceed recommended benchmarks, underscoring the need for further optimization and validation.

At the item level, predictors were associated with performance on gold-standard measures of language and reading skills, identifying children who appear to be at risk for dyslexia and DLD. Among the six items most predictive of identifying children at-risk for dyslexia, five were directly related to knowledge of letter-sound correspondence. Previous research has also found that letter-sound knowledge is a key predictor of children's reading development (5). For DLD, the most predictive items were indicators of poor performance on gold-standard measures of expressive and receptive language skills, suggesting that caregivers accurately identify these challenges in children when directly queried. The current study built on the work of Hendricks and colleagues by including behavioral examples as anchors within each item in an effort to help caregivers more reliably assess the constructs of interest, (22) which can be abstract.

Although Hendricks et al. found that caregivers may not be reliable at identifying signs of DLD in their child, the Hendricks et al. caregiver questionnaire did not include examples that could have helped caregivers identify specific areas of concern (22). We hope that by engaging caregivers early in the LDYSQ development to evaluate and refine item clarity, we strengthened caregivers’ understanding of each item and improved the measure's accuracy.

Measures for identifying bilingual children at risk of DLD or reading difficulties are also limited. Our preliminary results indicate that the questionnaire holds promise pending further validation. These results are consistent with those of Auza et al. and Restrepo who found that a caregiver questionnaire could lead to accurate identification of DLD (19, 21). Screeners for bilingual children at risk of reading difficulties are mostly available at the classroom level and were not developed for use in primary care settings. Therefore, findings related to bilingual participants should be interpreted as preliminary and hypothesis-generating rather than definitive.

Given the high prevalence and comorbidities of DLD and dyslexia (29) and the high rate of under identification in monolingual and bilingual populations, (22, 41) we must develop multiple systems to screen and prevent significant academic difficulties when left untreated and without early identification. Many states now require screening for reading difficulties, but not for DLD. The LDYSQ holds promise for integration into pediatric healthcare settings with further development and validation. With the success of pediatric healthcare initiatives like Bright Futures, screeners like the LDYSQ could contribute meaningfully to early identification of risk for dyslexia and DLD within pediatricians offices. Such screening could operate outside of overburdened educational systems, while still leveraging the resources available within schools to provide follow-up testing and connection with tutoring and additional support.

### Limitations and future directions

There are a few limitations to this study that warrant consideration. First, we did not collect demographic data from our participants, including their race, ethnicity, or socioeconomic status. This precludes us from generalizing our findings to a broader sample. Future research on the LDYSQ will include a more comprehensive demographic questionnaire to enable a more detailed description of our sample.

Another limitation of the current study was the small sample size, particularly among Spanish-English bilingual participants. This can lead to inflated estimates due to partial or complete separation, limiting our ability to assess the reliability of all items. It also limited our ability to conduct DIF analyses to ensure the measure performs equally well across monolingual and bilingual samples. Future work will include larger sample sizes for monolingual and Spanish-English bilingual groups. We aim to oversample at the low end of the distribution to identify the DLD-only risk group to address these concerns. Further, we will examine the instrument factors’ structure and invariance across groups in future studies.

Our pilot data from this study confirm the feasibility of the LDYSQ screener and its promise of achieving high overall accuracy in identifying children at risk for dyslexia and DLD. Currently, the sensitivity and specificity do not exceed the 80% benchmark across all outcomes. However, this is expected because the pilot study was designed to test the feasibility of the LDYSQ and evaluate its promise through an initial set of items, not to optimize the screener for broader use. The 14-item set is provisional; this will not be the finalized instrument. Our purpose is to show the feasibility of the measure and process and present preliminary evidence. A larger validation study will refine the item pool by using questions that are most sensitive to either disability, thereby improving the performance of the screener. We will use open science framework and register the validation study.

## Conclusion

Results from this pilot study show the feasibility and promise of the LDYSQ for improving risk assessment for dyslexia and DLD in 6-year-old English monolingual and Spanish-English bilingual children.

## Data Availability

The datasets presented in this article are not readily available because the authors do not have IRB approval to share the data. Requests to access the datasets should be directed to Dr. Restrepo marestrepo@usf.edu.
