# Supplementary material for: Development and item selection of the language and dyslexia screening questionnaire in primary care settings
Source: Front Pediatr. 2026 May 13;14:1750893. doi: 10.3389/fped.2026.1750893 (PMC13212353; doi:10.3389/fped.2026.1750893)
Supplement: Supplementary file 1 [file Table1.docx]

**Supplementary Table 1.** *Internal Consistency and Inter-Rater Reliability by Measure.*

|  | **Internal Consistency** | | **Inter-Rater Reliability** | |
| --- | --- | --- | --- | --- |
| **Measure** | N | Cronbach’s alpha^a^ (α) | N | Intraclass Correlation Coefficient (ICC) |
| **KBIT-2** | 149 | .878 | 12 | .997 |
| **CTOPP-2** |  |  |  |  |
| Elision | 143 | .940 | 12 | .867 |
| Blending Words | 144 | .910 | 12 | .998 |
| Sound Matching | 142 | .926 | 12 | .999 |
| Memory for Digits | 143 | .806 | 12 | .996 |
| Nonword Repetition | 139 | .786 | 12 | .989 |
| Rapid Digit Naming^b^ | - | - | 10 | .999 |
| Rapid Letter Naming^b^ | - | - | 12 | .999 |
| Rapid Color Naming^b^ | - | - | 12 | .999 |
| Rapid Object Naming^b^ | - | - | 12 | 1.00 |
| **WRMT-3** |  |  |  |  |
| Word Identification | 144 | .938 | 12 | .998 |
| Word Attack | 141 | .923 | 10 | .996 |
| **CELF-5^c^** |  |  |  |  |
| Sentence Comprehension | 110 | .818 | 11 | 1.00 |
| Word Structure | 110 | .757 | 11 | .982 |
| Formulated Sentences | 110 | .866 | 10 | .919 |
| Recalling Sentences | 110 | .913 | 10 | .993 |
| **BESA English^d^** |  |  |  |  |
| Morphosyntax Cloze Items^e^ | 32 | .936 | - | - |
| Sentence Repetition^e^ | 32 | .915 | - | - |
| Semantics^e^ | 31 | .893 | - | - |
| **BESA Spanish^d^** |  |  |  |  |
| Morphosyntax Cloze Items^f^ | 38 | .921 | - | - |
| Sentence Repetition^f^ | 38 | .964 | - | - |
| Semantics^f^ | 38 | .927 | - | - |
| **TOPPS^d^** |  |  |  |  |
| First Sound^f^ | 38 | .789 | - | - |
| Rapid Letter Naming^b, f^ | - | - | - | - |
| Memory for Digits^f^ | 38 | .726 | - | - |
| Blending Words^f^ | 36 | .877 | - | - |
| Segmenting Words^f^ | 35 | .852 | - | - |

Notes: ^a^Reported alpha is raw Cronbach's alpha; ^b^Internal consistency not psychometrically appropriate; ^c^Measure administered only to English monolingual participants; ^d^Measure administered only to English-Spanish bilingual participants; ^e^Inter-rater reliability unable to be calculated as only one participant was double-scored; ^f^Inter-rater reliability unable to be calculated as no participants were double-scored
